# A Case of Posttraumatic Pott’s Disease

**DOI:** 10.7759/cureus.26380

**Published:** 2022-06-27

**Authors:** Kyle Risos, Neel A Duggal, Shiwani Kamath, Adam Wolberg, Koravangala K Sundaresh

**Affiliations:** 1 Internal Medicine, HCA Florida Trinity Hospital, Trinity, USA; 2 Radiology, HCA Florida Trinity Hospital, Trinity, USA; 3 Infectious Diseases, HCA Florida Trinity Hospital, Trinity, USA

**Keywords:** spinal tuberculosis:, spinal trauma, tuberculosis spondylitis, skeletal tuberculosis, pott’s disease, tuberculosis

## Abstract

Tuberculosis infection, which is caused by the bacterium *Mycobacterium tuberculosis *(Mtb), most commonly manifests in patients with respiratory systems. However, it can also colonize other tissues including skeletal. In our case, a 77-year-old Caucasian male presented to the emergency department following a rollover motor vehicle collision with chief complaints of neck and lower back pain. After clinical improvement and a preliminary negative workup, the patient was deemed stable for discharge. Four months later, the patient was subsequently admitted for worsening back pain with workup suspicious for T9 and T10 discitis osteomyelitis and abscess formation on computed tomography (CT). During this admission, spinal Mtb was confirmed by acid-fast stain and real-time polymerase chain reaction of a CT-guided disc space aspirate of a left paraspinal cystic collection at approximately T9-T10. Given these findings, the patient was subsequently put on standard four-drug therapy for Mtb. Our case demonstrates the importance of considering Pott’s disease in the diagnosis of lumbar spinal pain, especially in patients living in areas with high international migration and travel.

## Introduction

Tuberculosis is caused by a bacterium called *Mycobacterium tuberculosis* (Mtb).* *These are non-motile, weakly-gram positive, acid-fast bacilli part of the order Actinomycetales​​​​​​ ​[1]. Mtb most commonly infects lung tissue leading to pulmonary symptoms such as productive cough, hemoptysis and shortness of breath. However, Mtb can infect other tissues of the body including bone. The most common site of skeletal Mtb infection is the spine [2]. Spinal Mtb, also known as Pott’s disease or tuberculosis spondylitis, results from the hematogenous spread of Mtb bacteria from an extra-spinal focus to the spine resulting in osteomyelitis or a cold abscess. The most common site of spinal Mtb infection is the thoracolumbar junction; however, any segment of the spine can be infected [3-6]. The most common presenting symptom of spinal Mtb infection is progressively worsening pain in the neck and spine over the span of weeks to months. In the majority of cases, chronic back pain is the only presenting symptom [7]. Chronic back pain is sometimes accompanied by muscle spasms and changes in posture and gait. Mtb infection often presents with constitutional symptoms such as weight loss, fever, and night sweats (especially if disseminated Mtb is present) [3,8].

Here, we report a case of a patient initially admitted with lower thoracic back pain following a motor vehicle collision found to have Pott's disease upon subsequent admission.

## Case presentation

**Figure 1:**
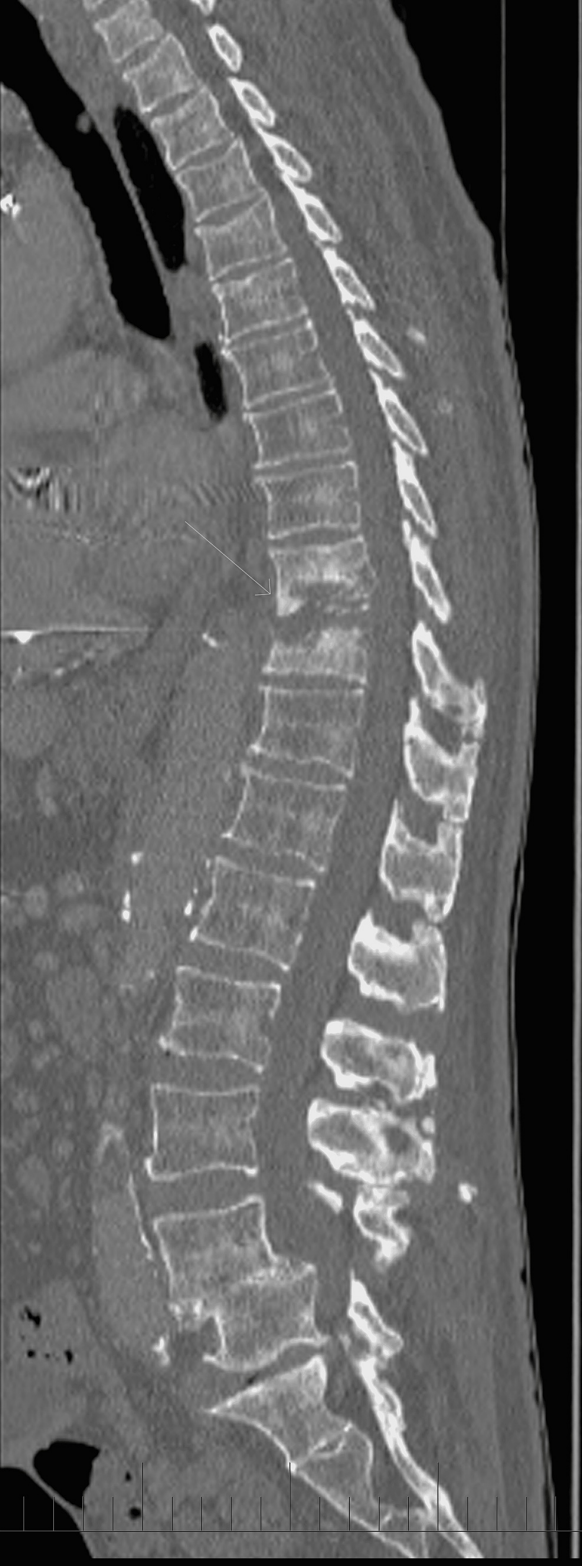
Computed tomography sagittal view of the thoracic spine from the initial visit This demonstrates bony destruction of the T9 and T10 endplates.

**Figure 2:**
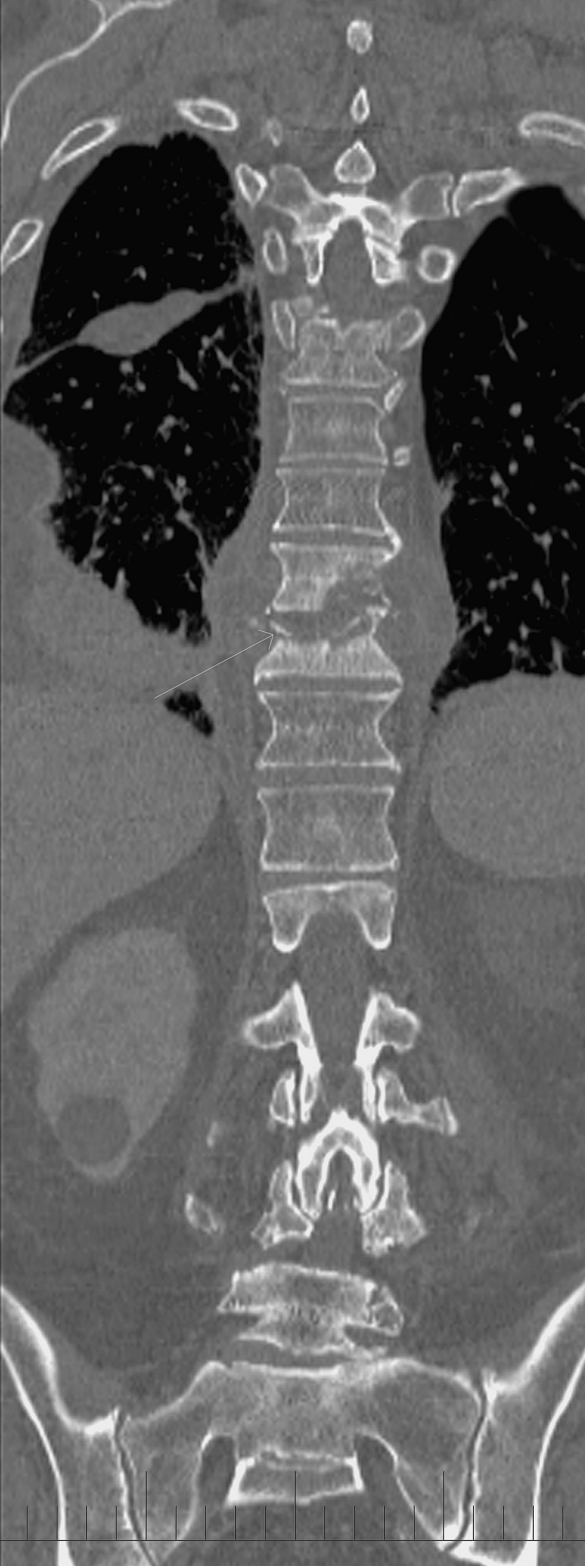
Computed tomography coronal view of the thoracic spine from the initial visit This demonstrates bony destruction of the T9 vertebral body.

A 77-year-old Caucasian male initially presented to the emergency department (ED) following a rollover motor vehicle collision with chief complaints of neck and lower back pain. The patient was driving while wearing his seatbelt and was under sternal precautions for a coronary artery bypass graft (CABG) five months prior for which he was taking clopidogrel and aspirin. Physical examination was noteworthy only for midline spinal tenderness to palpation throughout the cervical, thoracic, and lumbar spine without deformities or neurological deficits. A computed tomography (CT) scan of the entire spine revealed fracture of the second cervical vertebrae (C2), multiple fractures of the lumbar transverse processes, and a lucent spinal lesion of the eighth cervical vertebrae (C8) concerning for malignancy. A nuclear bone scan revealed increased uptake at the eighth and ninth thoracic (T8-T9) vertebrae that correlated with the lesions seen on the CT scans of the spine (Figures 1, 2).

The patient’s past medical history was extensive and included sick sinus syndrome with placement of permanent cardiac pacemaker, diabetes mellitus with associated nephropathy and neuropathy, hypertension, coronary artery disease, hypothyroidism, hyperlipidemia, and peripheral vascular disease with left third-toe amputation. The patient also reported distant early-stage bladder cancer that was initially treated with intravesical therapy followed by annual cystoscopy. He further reported a 120 pack-year smoking history and that he quit smoking 10 years ago. He noted a recent unintentional weight loss of 60 pounds over the prior 10 months without loss of appetite and chronic lumbar pain from a work injury about 33 years prior.

The patient was admitted for a trauma evaluation and his neck was immobilized and placed in a cervical collar. He was seen by the neurosurgeon who recommended no acute surgical intervention. CT-guided needle biopsy of the T9 paravertebral soft-tissue abnormality and core biopsy of the T9 vertebra were performed, revealing necrotizing granulomas with rare giant cells and no evidence of carcinoma or acid-fast bacteria; no mycobacteria were identified by Fite's stain. Kappa and lambda light-chain tests, serum levels of immunoglobulinIg G (IgG), IgA, and IgM, protein electrophoresis, and immunoelectrophoresis for the assessment of multiple myeloma were all within normal limits.

**Figure 3:**
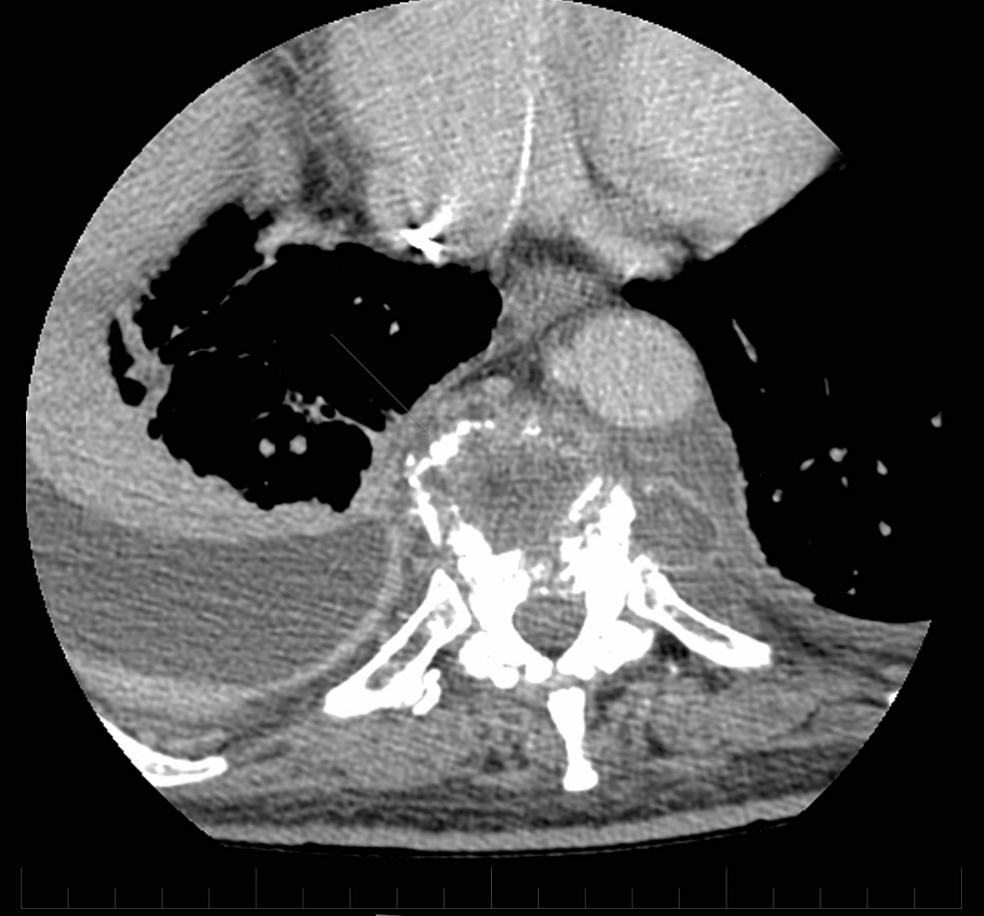
Computed tomography axial view of the thoracic spine during subsequent admission This demonstrates significant worsening bony destruction of the T9-T10 vertebral bodies involving the adjacent endplates. There is additional prevertebral soft tissue edema.

Four months later, the patient was seen at another ED due to worsening dull back pain in the lower thoracic area radiating to his left chest and left flank. He reported the pain as continuous since his motor vehicle collision without aggravating or alleviating factors, and that since his prior discharge, his pain was unsuccessfully controlled by his primary care and pain management physicians. Furthermore, he was not a surgical candidate for the spinal fractures he sustained from the collision. He denied saddle anesthesia and associated bowel or bladder symptoms. A pleural effusion suggestive of empyema in the right chest was found incidentally on chest CT without contrast. CT of the abdomen and pelvis without contrast revealed an abnormal disc space at T9-T10 with destruction of adjacent endplates suggesting acute discitis with osteomyelitis (Figure 3). A subsequent CT scan of the thoracic spine with contrast demonstrated T9-T10 discitis and osteomyelitis with abscess formation and significant loss of the T9 and T10 vertebral bodies.

He was admitted for further management, and pulmonology, hematology-oncology, neurosurgery, and infectious disease departments were consulted. When interviewed, the patient endorsed working for the U.S. Navy as a young adult, living in multiple regions of the USA and Germany throughout his life, and recreationally hunting wildlife.

Purified protein derivative (PPD), QuantiFERON, and Brucella serology were negative. Ultrasound-guided aspiration of the right-sided pleural effusion was positive for exudative fluid; cytology was negative for malignancy and insignificant for bacterial stains and culture. Because the patient was a poor surgical candidate for open biopsy of the lesion, a CT-guided disc space aspiration at approximately T9-T10 (which was the area of concern for discitis/osteomyelitis) was performed with drainage catheter placement. Acid-fast staining of the specimen was positive and the patient was started on a four-drug antituberculous treatment regimen of oral rifampin 600 mg/day, isoniazid 300 mg/day, pyrazinamide 500 mg thrice/day, and ethambutol 1200 mg/day (RIPE).

Real-time polymerase chain reaction (PCR) of the aspirate resulted positive for Mtb. The patient was diagnosed with clinically stable Pott’s disease. He tolerated the antituberculous treatment regimen well and was discharged with instructions to receive an ophthalmological evaluation within two weeks of discharge to monitor for optic neuropathy, weekly complete blood count (CBC) and complete metabolic panel (CMP) for four weeks and then monthly until completing drug regimen, and to continue the RIPE drug regimen for two months followed by the rifampin/isoniazid drug regimen for six months.

## Discussion

Pott’s disease is relatively uncommon in high-income countries such as the United States [7]. Thus, it is not often considered when diagnosing spinal pathologies. Diagnosis of spinal Mtb infection in the United States is often delayed due to its subacute presentation and low incidence of Mtb [3,9]. Other challenges in diagnosing spinal Mtb infection include lack of specific clinical diagnostic criteria and preemptive ruling out of Mtb before further confirmation with biopsy or nucleic acid amplification testing (NAAT) [10]. In our patient, Pott’s disease as a cause of his neck and back pain was reasonably considered but initially unconfirmed. In addition, acid-fast stains and Mycobacterium culture from the initial admission were negative. Data indicates that false negative results for Mtb infection are relatively common with acid-fast stains [11].

Several case reports have documented Pott’s disease in patients where other diagnoses were initially pursued [12-15]. The majority of these patients had significant risk factors for Mtb infection including being immunocompromised and emigration from an endemic area. Our patient did have significant known risk factors such as diabetes mellitus and extensive international travel history. As such, it is uncertain if his trauma played a role in the manifestation of Pott’s disease or simply aggravated a preexisting infection.

Diagnosis of spinal tuberculosis is multifaceted and includes components of clinical history, laboratory results, and imaging findings [16]. As most spinal infections typically come from a pulmonary focus or extra-pulmonary foci such as lymph nodes, a thorough review of systems can help tease out the underlying source of infection [16]. Although our patient did not have any pulmonary symptoms, imaging revealed a pleural effusion. A comprehensive evaluation of other risk factors, such as being born prior to the 1950s, poverty, traveling or living in endemic regions or areas of overcrowding, imprisonment, drug abuse, alcohol abuse, malnutrition, diabetes mellitus, chronic peritoneal dialysis, human immunodeficiency virus infection, other immunosuppressive states, and prior tuberculosis infection, is key in establishing the diagnosis [7].

The initial laboratory workup for Mtb infection should include a PPD skin test and CBC; if a PPD cannot be done or is inconclusive, a QuantiFERON Mtb gold test (a form of enzyme-linked immunospot assay) should be obtained. Bodily fluids and pus from abscesses should undergo testing for acid-fast bacteria. Biopsies of solid lesions should undergo PCR testing and evaluation for granulomas and caseating necrosis. The gold standard of laboratory testing for Mtb is Mycobacterium cultures from collected fluids or samples. Magnetic resonance imaging is the image of choice for spinal Mtb infection and typically shows lesions that originate from the vertebral endplate, vertebral body collapse or destruction, paraspinal abscess formation, or abscess calcification [16].

Delayed diagnosis of Pott’s disease can lead to detrimental outcomes including degenerative spinal stenosis and paralysis [10]. Despite a considerable decline in the incidence of Mtb infection within the United States, there were still 7174 reported cases in 2020 [17]. The literature suggests that Mtb infection itself is increasing in prevalence in higher income countries due to increased global migration [18].

Because of the relatively high proportion of adult patients with Mtb infection caused by drug-resistant Mtb, four drugs are necessary in the initial phase for the six-month regimen to be maximally effective [19]. In most circumstances, the treatment regimen for all adults with previously untreated tuberculosis should consist of a two-month initial phase of RIPE as used in our patient [19]. Surgical treatment is considered in cases of severe spinal instability or progressive neurological symptoms with evidence of cord compression or deformation [3]. Finally, the prognosis of Pott's disease depends on many factors. There is more likely to be a better prognosis if the patient is young, without comorbidities, only has partial cord compression, has early-onset cord involvement, and exhibits slow and short duration of neural complications [3].

## Conclusions

Pott’s disease is a relatively unusual complication of infection with *Mycobacterium tuberculosis*. However, it should be considered in the diagnosis of spinal pain in patients with significant risk factors and accompanying history such as travel and unexplained weight loss. Initial smearing for acid-fast bacilli may be negative, but it is important to confirm the diagnosis with biopsy results. Early management of Pott's disease is crucial to preventing long-term complications of the disease and improving prognosis.
